# Effects of diabetes mellitus complicated by admission hyperglycemia on clot histological composition and ultrastructure in patients with acute ischemic stroke

**DOI:** 10.1186/s12883-022-02660-y

**Published:** 2022-04-05

**Authors:** Qun Gao, Peng Qi, Junjie Wang, Shen Hu, Ximeng Yang, Jingwen Fan, Ling Li, Yao Lu, Jun Lu, Juan Chen, Daming Wang

**Affiliations:** 1 Department of Neurosurgery, Beijing Hospital, National Center of Gerontology, No.1 DaHua Road, Dong Dan, Beijing, 100730 People’s Republic of China; 2 Graduate School of Peking Union Medical College, Beijing, China; 3 Peking University Fifth School of Clinical Medicine, Beijing Hospital, Beijing, China; 4 Department of Radiology, Beijing Hospital, National Center of Gerontology, Beijing, China; 5 Beijing Institute of Geriatrics, Beijing Hospital, National Center of Gerontology, Beijing, China

**Keywords:** Thrombus, Type 2 diabetes mellitus, Admission hyperglycemia, Ultrastructure, Clot perviousness

## Abstract

**Background:**

Type 2 diabetes mellitus (T2DM) affects the occurrence and prognosis of acute ischemic stroke (AIS). However, the impact of diabetes on thrombus characteristics is unclear. The relationship between the composition and ultrastructure of clots and DM with admission hyperglycemia was investigated.

**Methods:**

Consecutive patients with AIS who underwent endovascular thrombus retrieval between June 2017 and May 2021 were recruited. The thrombus composition and ultrastructure were evaluated using Martius scarlet blue stain and scanning electron microscopy. Clot perviousness was evaluated via thrombus attenuation increase on computed tomography angiography (CTA) versus non-contrast CT. Patients with admission hyperglycemia DM (ahDM) and those without DM (nonDM) were compared in terms of thrombus composition, ultrastructure, and perviousness.

**Results:**

On admission, higher NIHSS scores (17 vs. 12, respectively, *p* = 0.015) was evident in ahDM patients. After the 90-day follow-up, the rates of excellent outcomes (mRS 0–1) were lower in patients with ahDM (16.6%, *p* = 0.038), but functional independence (mRS 0–2) and handicapped (mRS 3–5) were comparable between patients with ahDM and nonDM. The outcome of mortality was higher in patients with ahDM (33.3%, *p* = 0.046) than in nonDM patients. Clots in patients with ahDM had more fibrin (39.4% vs. 25.0%, respectively, *p* = 0.007), fewer erythrocyte components (21.2% vs. 41.5%, respectively, *p* = 0.043), equivalent platelet fraction (27.7% vs. 24.6%, respectively, *p* = 0.587), and higher WBC counts (4.6% vs. 3.3%, respectively, *p* = 0.004) than in nonDM patients. The percentage of polyhedral erythrocytes in thrombi was significantly higher in ahDM patients than in nonDM patients (68.9% vs. 45.6%, respectively, *p* = 0.007). The proportion of pervious clots was higher in patients nonDM than in patients with ahDM (82.61% vs. 40%, respectively, *p* = 0.026).

**Conclusion:**

Patients with ahDM presented with greater stroke severity on admission and poorer functional outcomes after 3 months. Clots in patients with ahDM had more fibrin, leucocytes, and fewer erythrocyte components than in patients nonDM. The content of polyhedral erythrocytes and impervious clots proportion were significantly higher in thrombi of patients with AIS and ahDM. Further research is required to validate these findings.

## Background

Acute ischemic stroke (AIS) is the most common cause of mortality and long-term disability worldwide [1]. The risk of AIS is more than two-fold higher and more severe in patients with type 2 diabetes mellitus (T2DM) [2]. It is associated with poorer functional outcomes and higher mortality risk [3]. About 40 to 60% of patients with AIS present with admission hyperglycemia either due to acute stress response or diabetes [4]. In both diabetics and non-diabetics AIS patients, hyperglycemia at the time of admission has been associated with negative outcomes [5]. It is related to the stress response of AIS patients as a result of excessive secretion of steroid hormones, adrenaline, glucagon and free fatty acids [6]. Furthermore, diabetes mellitus and acute hyperglycemia could enhance oxidative stress and inflammation response, impair cerebrovascular reactivity in the microvasculature, provoke a prothrombotic state, and cause cerebral injury [7].

The development of mechanical thrombectomy (MT) has enabled investigations of the composition and structure of human cerebral thrombi [8]. Cerebral thrombi consist of four major components: red blood cells (RBCs), fibrin, platelets, and white blood cells (WBCs) [9]. The evaluation of retrieved clots from patients with AIS may improve our knowledge of stroke pathology and predict treatment response. RBC-rich thrombus might be easier to recanalize in patients with AIS, while fibrin-rich clots are more refractory [10]. The detailed examination of the thrombi can help determine the effectiveness of various treatment approaches for patient selection.

Depending on the clot ultrastructure, most cerebral clots undergo intravital thrombus contraction (retraction), which may be clinically significant. In blood clots, activated platelets produce contractile forces transferred via the fibrin network [11], creating a platelet-fibrin meshwork that accumulates at the periphery of the clots and compresses RBCs into the center of the clot [12]. RBCs are one of the most abundant components of cerebral thrombi. Clot contraction leads to a reduction in the thrombus volume and deformation of the RBCs, including polyhedrocytes and polyhedral RBCs, which comprise the majority of RBCs. Polyhedrocytes provide an impermeable seal because of minimal interstitial space, promoting fibrinolysis resistance [12, 13]. Much research has shown the hyper-reactivity of platelets from diabetic patients, as evidenced by increased fibrinogen binding and enhanced aggregation [14]. In addition, acute hyperglycemia in T2DM can promote further platelet activation [15]. However, the effects of ahDM on forces generated by clot contraction on RBCs have not been investigated.

Although the negative effects of T2DM on cerebrovascular reactivity and reperfusion damage are well established, the effects of DM on the composition and ultrastructure of thrombi in AIS remain unclear. A previous study showed that clots in patients with DM had more fibrin and fewer RBC components than in nonDM patients, while hyperglycemia on admission did not show an association with clot composition [16]. The present study aimed to evaluate the association between ahDM and the composition and ultrastructure of clots in patients with AIS.

## Materials and methods

Consecutive patients with AIS who underwent MT at Beijing Hospital between June 2017 and May 2021 were enrolled. Inclusion criteria were as follows: (1) AIS caused by an occlusive intracranial clot (both anterior and posterior circulation); (2) availability of data about preoperative computed tomography (CT) (non-computed CT [NCCT] and CT angiography [CTA]) evaluation; and (3) suitable clots retrieved from patients who had undergone MT for histopathological and ultrastructural analysis. Diabetes mellitus complicated by admission hyperglycemia (ahDM) was defined as a history of physician-diagnosed T2DM with a plasma glucose level > 7.80 mmol/L in a random state when admitted to hospital caused by AIS [17]. Non-DM AIS patients were defined as patients without DM and with normal plasma glucose (≤ 7.80 mmol/L) on admission. All study participants provided informed consent. The Beijing Hospital Ethics Committee (2019BJYYEC-130-01) approved this study as it met national and international guidelines for research on humans.

### Clinical data collection and assessment

Demographic features (age and sex), medical history (hypertension, dyslipidemia, glycemia on admission, smoking history, atrial fibrillation, coronary artery disease, and stroke or transient cerebral ischemia), clinical and laboratory data, anticoagulant and/or antiplatelet use, thrombus location (the first segment of the middle cerebral artery [M1], second segment of the middle cerebral artery [M2], anterior cerebral artery [ACA], terminal internal carotid artery [ICA], and basilar artery [BA]), and procedural characteristics were recorded for analysis. Stroke severity was assessed using the National Institutes of Health Stroke Scale (NIHSS) score. Causes of stroke were classified using the Trial of ORG 10172 in Acute Stroke Treatment criteria [18]. Procedural and clinical outcomes were MT strategy (contact aspiration [CA], stent retriever [SR], and combination techniques), the number of maneuvers, and revascularization outcomes including complete reperfusion [eTICI2c-3] after completion of the procedure [19]. Outcomes were assessed 3 months post-MT using the modified Rankin scale (mRS) score obtained by outpatient or telephonic follow-up. Clinical outcomes were divided into excellent outcome (mRS 0–1), functional independence (mRS 0–2), handicapped (mRS 3–5), and mortality (mRS 6).

### Histological staining

The retrieved thrombi were immediately washed with phosphate-buffered saline for several minutes and fixed in a fixation buffer. The samples were sectioned longitudinally to observe the overall condition of the thrombi. The clots were embedded in paraffin, cut in 4 mm sections, and stained using Martius scarlet blue (MSB). Based on the MSB staining results, the proportion of each component (fibrin, RBCs, WBCs, and platelets) was quantified using Orbit Imaging Analysis machine-learning software (www.Orbit.bio, Idorsia Ltd.) [20].

### Scanning electron microscopy

Longitudinally sectioned clots were serially dehydrated in an ethanol gradient (10 min each in 50, 70, 95, and 100% ethanol). The samples were subsequently dried in carbon dioxide, fixed to a colloidal carbon stubble, and sputtered onto the surface using a sputtering device. The samples collected were inspected using a scanning electron microscope (SEM; JEOL 7500) at the University of Peking’s medical department. At least five images were analyzed for each thrombus. Images were obtained in randomly selected areas between the edge and center of the thrombus. Quantitative assessment of the RBCs of the thrombi was performed manually using the Image J software (Bethesda, MD, USA), as previously described [21]. A grid (4 μm × 4 μm) was briefly overlaid on the scanning electron images. Each grid square is approximately the size of a cellular structure. A grid square usually contains either the whole structure or a part of it. The number of complete or partial squares (4 μm × 4 μm) occupied by each structural component, if there were multiple structural components, was computed for the whole sample based on all images. The number of squares occupied by each structural component was added and then divided by the total number of squares analyzed for all structural components within the sample to obtain the fraction of the area occupied by each structural component.

### Measurement of imaging parameters

A 320 × 0.5 mm detector row CT scanner (Aquilion ONE, Canon Medical Systems) was used for imaging evaluation on admission. All patients underwent NCCT and CTA. Based on NCCT and related CTA images, the increase in thrombus attenuation in the regions of interest within each clot was evaluated to determine the degree of clot permeability. The mean Hounsfield unit (HU) values of the thrombus on NCCT and CTA were recorded as HU_CT_ and HU_CTA_, respectively. The absolute clot perviousness (δHU) was calculated as δHU = HU_CTA_-HU_CT_. Pervious clots were defined as δHU ≥ 10 HU, whereas impervious clots were defined as δHU < 10 HU.

### Statistical analysis

Variations in baseline characteristics, procedural results, and clinical outcomes were examined between patients with ahDM and nonDM. The normality of continuous variables was assessed using the Kolmogorov-Smirnov test. Normally distributed variables were expressed as mean ± standard deviation, and differences were analyzed using the Student’s t-test. Non-normally distributed variables were expressed as median (interquartile range [IQR]), and differences were analyzed using the Mann-Whitney U test. Categorical variables were presented as counts (percentages), and differences were analyzed using the Fishers exact test. The significance threshold for all tests was set at *p* < 0.05. GraphPad Prism 8 software was used for all statistical analyses.

## Results

### Baseline characteristics

A total of 55 patients (age, 76 (IQR 62–85) years; 38 men) were included, and 30 were diagnosed with DM and admission hyperglycemia. The clinical and laboratory characteristics of the patients are presented in Table 1. Patients diagnosed with ahDM had higher serum glucose on admission (12.9 vs. 5.5%, respectively, *p* < 0.001) and more severe stroke (NIHSS score, 17 [IQR, 9–24], *p* = 0.015) than nonDM patients. Other comorbidities were comparable between patients with and without ahDM. Stroke etiology was as follows: large artery atherosclerosis (15, 27.2%), cardiogenic embolism (34, 61.8%), cryptogenic stroke (3, 5.45%), and other (3, 5.45%). Thrombus location was in the ICA in 18 (32.7%), M1 in 18 (32.7%), M2 in 9 (16.3%), ACA in 2 (3.6%), and BA in 8 (14.5%) patients respectively. No significant differences were observed in thrombus location between patient groups (*p* > 0.05). Preoperative use of anticoagulants and antiplatelets was noted in 9 (16.4%) and 29 (52.7%) patients, respectively. No significant differences were observed in the laboratory evaluation of coagulation function (including APTT, PT, fibrinogen, INR, and D-dimer) on admission between patients with ahDM and nonDM.

**Table 1:** Baseline characteristics of ahDM and nonDM patients

	All patients *N* = 55	ahDM *n* = 30	non-DM *n* = 25	*p*
**Demographics**				
Age, y	76 (62–85)	76 (60–84)	72 (61.5–82.5)	0.818
Sex, male	38 (64.4%)	19 (63.3)	18 (72)	0.571
**Comorbidities**				
Atrial fibrillation	30 (54.5%)	16 (53.3)	14 (56)	0.99
Hypertension	40 (72.7%)	21 (70)	19 (76)	0.763
Dyslipidemia	31 (56.4%)	20 (66.6)	11 (44)	0.11
Stroke or TIA history	24 (43.6%)	13 (43.3)	11 (44)	0.99
Smoking history	30 (54.5%)	15 (50)	15 (60)	0.588
Coronary artery disease	25 (45.5%)	16 (53.3)	9 (36)	0.278
NIHSS	15 (9–20)	17 (9–24)	12 (6.5–16)	0.015
Serum glucose	8.2 (5.6–13.3)	12.9 (9.6–16.15)	5.5 (5.2–6.6)	< 0.001
**Medication**				
Anticoagulant use	9 (16.4%)	5 (16.7)	4 (16)	0.99
Antiplatelet use	29 (52.7%)	16 (53.3)	13 (52)	0.99
**Laboratory evaluation**				
APTT (s)	33.01 ± 4.54	32.64 ± 4.63	33.4 ± 4.41	0.541
PT (s)	11.2 (10.68–12)	11.1 (10.48–11.38)	11.4 (10.8–12.2)	0.209
Fibrinogen (g/L)	3.13 (2.58–3.49)	3.08 (2.57–3.55)	3.15 (2.71–3.45)	0.615
INR	0.97 (0.93–1.04)	0.97 (0.91–1.0)	0.99 (0.94–1.06)	0.234
D-dimer (ng/mL)	239.5 (153.8–621.5)	231 (161.8–519.8)	369 (137.0–653.5)	0.227
**Stroke etiology**				0.94
CE	34 (61.8)	18 (60)	16 (64)	
LAA	15 (27.2)	8 (26.6)	7 (28)	
Other determined	3 (5.45)	2 (6.7)	1 (4)	
Cryptogenic	3 (5.45)	2 (6.7)	1 (4)	
**Thrombus location**				0.623
ICA	18 (32.7)	10 (33.3)	8 (26.6)	
M1	18 (32.7)	8 (26.2)	10 (33.3)	
M2	9 (16.3)	5 (16.7)	4 (16)	
ACA	2 (3.6)	2 (6.6)	0 (0)	
BA	8 (14.5)	5 (16.6)	3 (12)	

*Notes*: Results are presented as median (IQR), number (percentage), or mean ± SD

*Abbreviations*: *TIA* transient ischemic attack, *NIHSS* National Institutes of Health Stroke Scale, *APTT* activated partial thromboplastin time, *PT* prothrombin time, *INR* international normalized ratio, *CE* cardiogenic embolism, *LAA* large artery atherosclerosis

### Procedural and clinical outcomes

Table 2 illustrates the differences in procedural and clinical results between patients with ahDM and nonDM. Treatment strategies were classified as SR (16, 29.1%), CA (21, 38.2%), and Solumbra (30, 54.5%). The median number of thrombectomy maneuvers was 2 (IQR, 1–3). After conclusion of the procedure, 22 (73.3%) patients with ahDM and 21 (84.0%) patients with nonDM underwent eTICI2c-3 recanalization. At the 90-day follow-up, 16 (29.1%) patients achieved excellent outcomes (mRS 0–1). The proportion of excellent outcomes was lower in patients with ahDM than in nonDM patients (16.6% vs. 44%, respectively, *p* = 0.038). Although 23 (41.8%) achieved functional independence (mRS 0–2) and 20 (36.4%) achieved handicapped outcomes, no significant difference was observed in the rates of functional independence and handicapped outcomes between the two groups. The mortality outcomes were significantly different between patients with ahDM and nonDM (33.3 and 8%, respectively; *p* = 0.046).

**Table 2:** Procedural and clinical outcomes of ahDM and nonDM patients

	All patients (*N* = 55)	ahDM (*n* = 30)	nonDM (*n* = 25)	*p*
**Strategy**				0.603
Stent retriever	16 (29.1)	11 (36.7)	5 (20)	
Contact aspiration	21 (38.2)	11 (36.7)	10 (40)	
Solumbra	30 (54.5)	18 (60)	12 (48)	
Number of maneuvers	2 (1–3)	2 (1–2.25)	2 (1.25–3)	0.422
eTICI2c-3	43 (78.2)	22 (73.3)	21 (84)	0.514
**Clinical outcomes**				
Excellent outcome	16 (29.1)	5 (16.6)	11 (44)	0.038
Functional independence	23 (41.8)	9 (30)	14 (56)	0.061
Handicapped	20 (36.4)	11 (36.7)	9 (36)	0.99
Mortality	12 (21.8)	10 (33.3)	2 (8)	0.046

*Notes*: Results are presented as number (percentage) or median (IQR)

*Abbreviations*: *CA* contact aspiration, *SR* stent retriever, Solumbra, combination of stent retriever and aspiration

### Histological composition

**Fig. 1:**
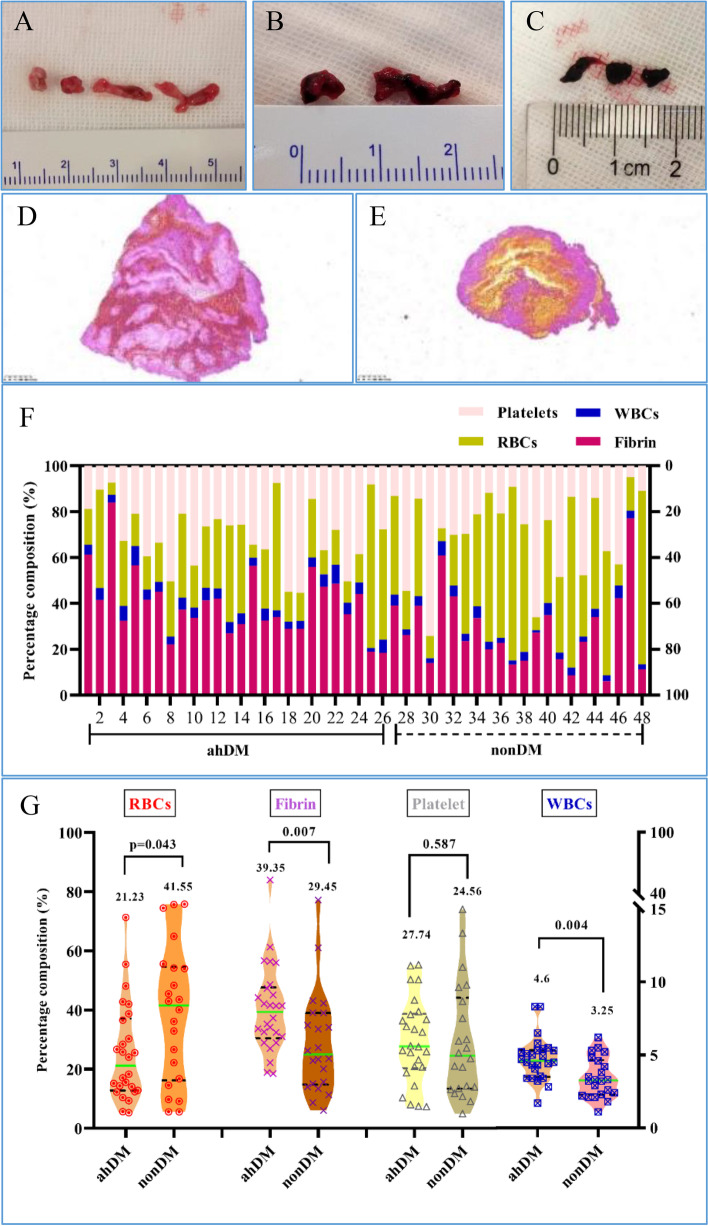
The appearance and composition of clots. Macroscopic images of typical retrieved clots classified into pinkish (**A**), red (**B**), and dark red (**C**). Representative clots from patients with ahDM (**D**) and nonDM (**E**) were stained using MSB to visualize the RBCs (yellow), fibrin (dark pink to red), WBCs (blue), and platelets (gray). Scale bar (MSB) = 200 μm. **F** Representation of the histological clot composition of each patient in the cohort as determined by MSB staining. **G** Violin plots display the differences in clot composition (RBCs, fibrin, platelets, and WBCs) according to ahDM history of DM or absence of DM on admission. ahDM, admission hyperglycemia diabetes mellitus; MSB, Martius scarlet blue; RBC, red blood cell; WBC, white blood cell

Based on RBC contents, the gross pathology of the retrieved thrombi was divided into three categories: pinkish, red, and dark red (Fig. 1A-C). MSB staining was used to characterize clot composition. Representative clot images in Fig. 1D and E illustrate a fibrin-rich clot in a patient with ahDM and an RBC-rich clot in a patient with nonDM. Analyses were performed on 48 of the 55 thrombi collected. Seven thrombi were not analyzed because of failed MSB staining. Assessment of the clot using MSB staining revealed heterogeneous composition of major clot components in the patient cohort (Fig. 1F). Compared with those in patients with nonDM, thrombi in patients with ahDM had more fibrin (25.0% vs. 39.4%, respectively, *p* = 0.007), fewer RBCs (41.5% vs. 21.2%, respectively, *p* = 0.043), and equivalent platelet content (24.6% vs. 27.7%, respectively, *p* = 0.587). Although the WBC content of clots was minimal, significant differences were observed between patients with ahDM and with nonDM (4.6% vs. 3.3%, respectively, *p* = 0.004) (Fig. 1G).

### SEM of RBCs

**Fig. 2:**
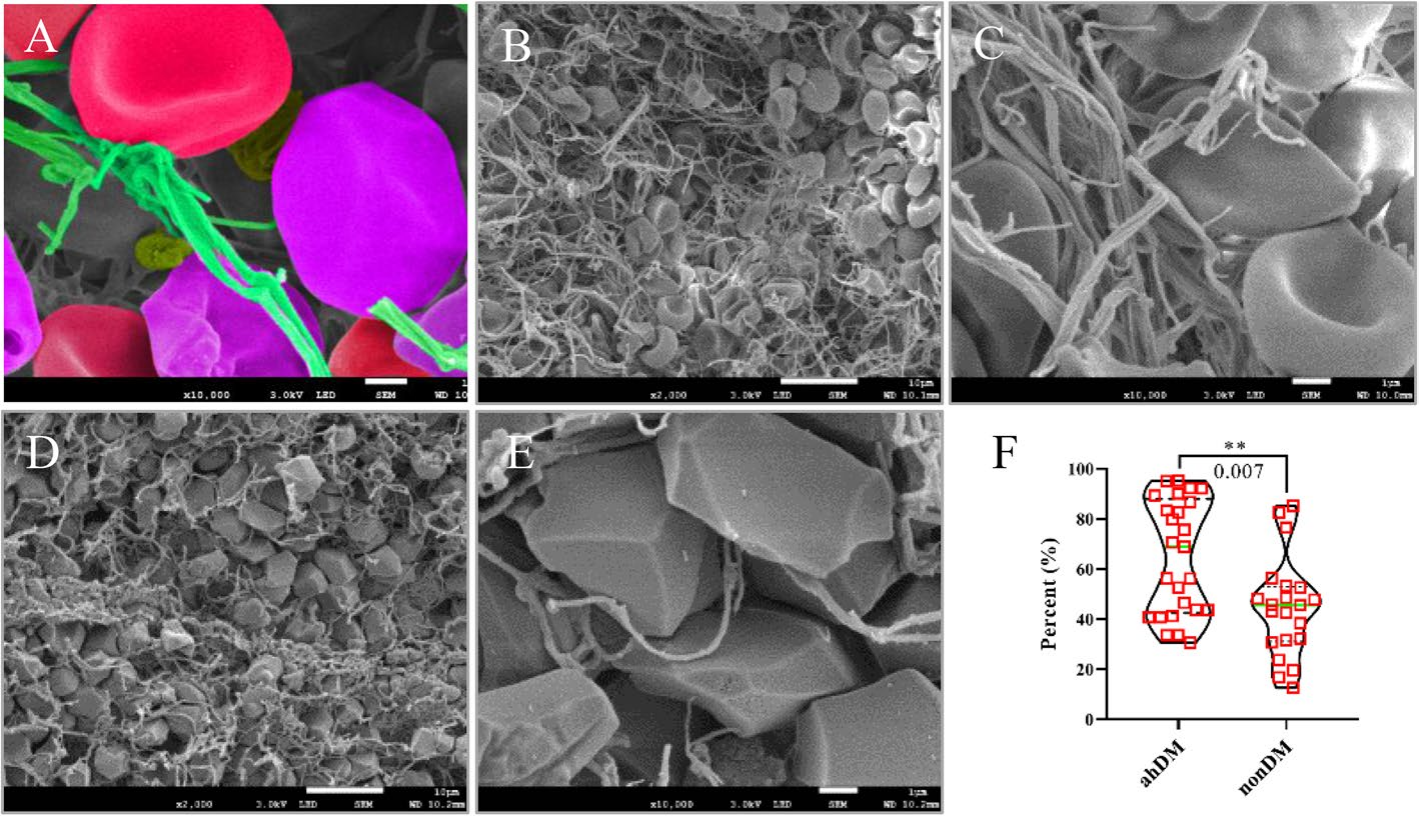
Analyses of structures of thrombi. **A** Selected colored portions of SEM images of thrombi illustrating the types of blood cells analyzed in this study: biconcave RBCs or predominantly biconcave intermediate-shaped RBCs (red); predominantly polyhedral intermediate-shaped RBCs or polyhedral compressed RBCs (polyhedrocytes) (purple); platelets (yellow); fibrin (green). Representative SEM visualization of thrombi ultrastructure in patients without DM (**B**, **C**) and patients with ahDM (**D**, **E**). Scale bar (SEM) = 10 μm (**B**, **D**); 1 μm (**C**, **E**). **F** Comparison of polyhedral RBC (polyhedrocytes) content in patients with ahDM and without DM. ahDM, admission hyperglycemia diabetes mellitus; SEM, scanning electron microscopy; RBC, red blood cell

RBC surfaces were examined using SEM. Biconcave cells either had a distinct concave structure or a side view of the circular portion of the biconcave cells. Contracted blood clots developed a notable structure, polyhedrocytes exhibited clearly defined polygonal faces, and the type of polygon was distinguishable (Fig. 2A). Thrombi in nonDM patients exhibited normal biconcave RBC morphologies, and RBCs were scattered in a disordered manner among the fibrin (Fig. 2B, C). In thrombi of patients with ahDM, RBC morphologies lacked double concave discs and adopted compressed polyhedral morphologies (Fig. 2D, E). The proportion of polyhedrocytes was higher in patients with ahDM than in nonDM patients (Fig. 2F, 68.9% vs. 45.6%, respectively, *p* = 0.007).

### Clot permeability

**Fig. 3:**
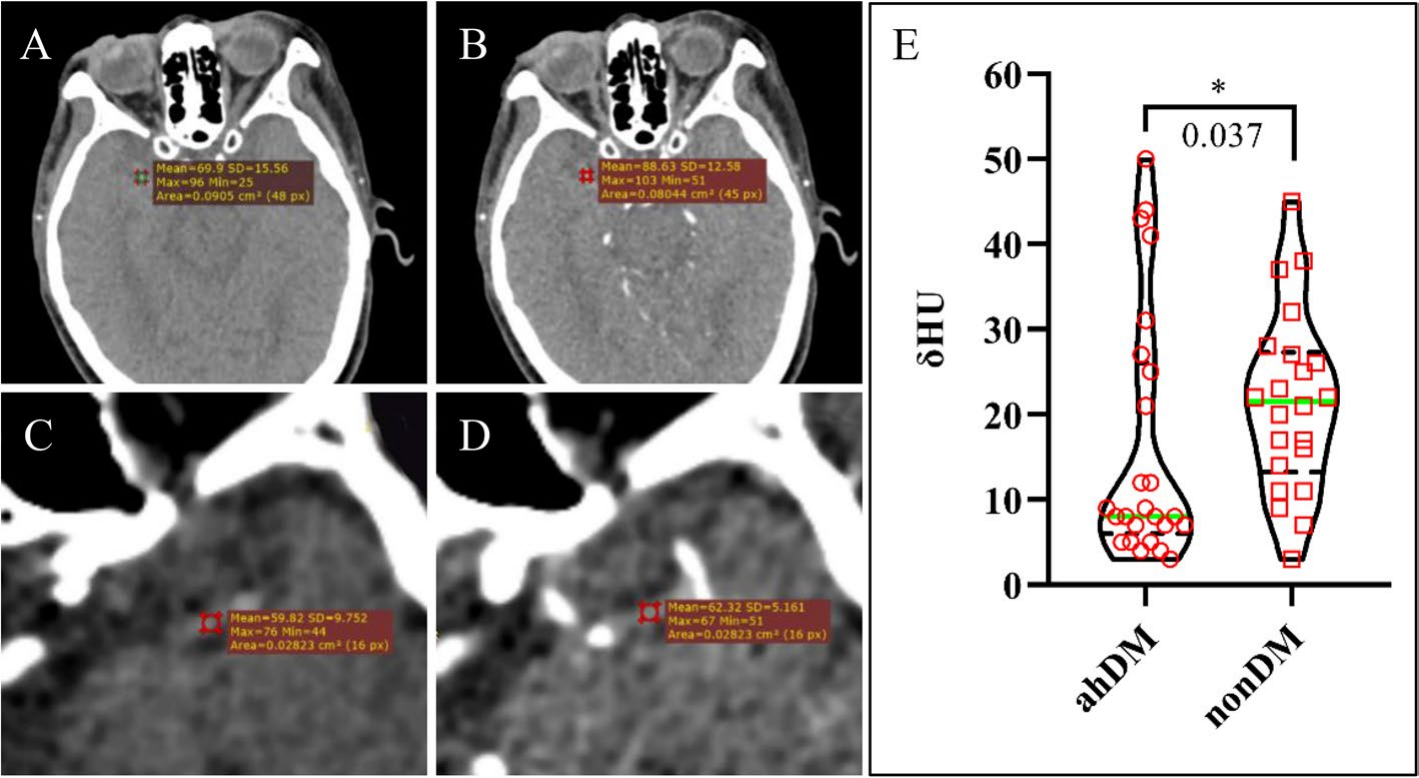
Clot perviousness measurement. Clot permeability was assessed using CT and CTA. Acquired non-contrast CT (**A**) and CTA (**B**) images of a representative patient without DM, and digitally zoomed (**C**, **D**) representative images of a patient with ahDM depict an occlusive clot in the M1 segment. Regions of interest of the clot assessed on non-contrast CT (**A**, **C**) and CTA (**B**, **D**). **E** δHU was significantly lower in patients with ahDM than in patients without DM. CT, computed tomography; CTA, computed tomography angiography; ahDM, admission hyperglycemia diabetes mellitus; δHU, Absolute thrombus perviousness; M1, first segment of the middle cerebral artery

Clot perviousness (or permeability) is a key imaging marker that is typically evaluated as an increase in HU values on CTA relative to those on NCCT. We compared clot permeability-based CT and CTA (Fig. 3a-d) between patients with AIS, ahDM and nonDM. Further, we determined the pervious nature of the clots by calculating the thrombus attenuation increase (δHU). δHU was significantly lower in clots of patients with ahDM than in nonDM patients (Fig. 3e, 8 (IQR, 6–2), *p* = 0.037). About dichotomous variables, patients with ahDM had a lower proportion of pervious clots (δHU ≥ 10 HU) than nonDM patients (40% vs. 82.61%, respectively, *p* = 0.026).

## Discussion

Analysis of the clinical characteristics and thrombi of 55 patients with AIS revealed that ahDM affected ischemic stroke severity and was associated with poorer functional outcomes. Clots of patients with ahDM had more fibrin, fewer RBCs, higher WBC counts, and an equivalent fraction of platelets compared to nonDM patients. Additionally, the proportion of polyhedrocytes in clots was higher, and that of pervious clots was lower in patients with ahDM than in patients without DM.

In the study, patients with ahDM presented with severe ischemic stroke. Patients with ahDM had poorer functional outcomes and higher 90-day mortality rates than nonDM patients. Previous studies have compared stroke severity between patients with T2DM and without T2DM, but conflicting results have been reported [22–25]. One study reported that stroke was more severe in patients with T2DM, which is consistent with our findings [22]. Furthermore, T2DM has been reported to independently predict more unfavorable functional outcomes at hospital discharge, whereby AIS patients with diabetes exhibit a three-fold higher mortality rate than patients without diabetes [26]. However, other studies have reported no association between T2DM and stroke severity or that patients with T2DM have a mild stroke on hospital admission [23–25]. Similarly, a previous study did not identify a significant difference in stroke severity between patients with T2DM and without T2DM [16]. Additionally, admission hyperglycemia of acute ischemic stroke causes increased ischemic injury via endothelial dysfunction, oxidative stress, and impaired fibrinolysis [27]. The patients with T2DM included in this study had admission hyperglycemia, partly explaining the discrepancy.

This study demonstrated that clots in patients with AIS and ahDM had fewer RBCs, more fibrin, equivalent platelets, and higher WBC counts than those with AIS and nonDM. Diabetes is characterized by hyperglycemia and insulin resistance, enhanced oxidative stress, inflammatory responses, activation of coagulation and platelets, and endothelial cell dysfunction. Hyperglycemia and insulin resistance can lead to elevated expression and secretion of plasminogen activator inhibitor-1 (PAI-1) [28]. PAI-1 inhibits fibrinolysis in thrombi predominantly by inhibiting plasminogen activator, which promotes fibrin degradation in thrombi. Moreover, glycosylated plasminogen in diabetes directly affects fibrinolysis by reducing plasmin generation and impairing functional protein activity, resulting in impaired fibrinolysis [29]. Increased plasma PAI-1 and glycation of plasminogen may be a potential mechanism underlying elevated fibrin content in the thrombi of patients with ahDM.

This study showed that platelet fraction was comparable between patients with and without DM and may be related to the effect of diabetes on platelets, which is centered on platelet activity [30]. P-selectin and GPIb/CD41 levels are elevated in patients with DM, indicative of platelet activation [31]. Patients with DM who experience myocardial infarction exhibit increased thrombin production and platelet activation [32]. Further, patients with diabetes are characterized by accelerated platelet consumption/production and a resultant increase in immature platelets [33]. Results demonstrated that WBC counts were higher in patients with ahDM than in nonDM patients. It is associated with stimulating oxidative stress and inflammation caused by ahDM. WBCs and platelets from patients with diabetes have been reported to be hyperreactive and express more adhesion molecules [34]. Additionally, activated platelets induce increased formation of circulating platelet-leukocyte aggregates [35].

The history of ahDM may provide clues regarding thrombus composition and facilitate decision-making to develop strategies for MT. A previous study reported that thrombolysis was less effective in thrombi with a high fibrin content than RBC-rich thrombi. In contrast, thrombi with a high RBC count were associated with successful reperfusion [36]. RBC-rich clots are easier to recanalize, whereas fibrin-rich clots are more difficult to recanalize in patients with AIS [10]. Thrombi have higher fibrin content, which increases friction with the vessel wall and makes it more difficult to remove the clot [37]. Therefore, recanalization of thrombi may be more difficult in patients with ahDM. However, we did not observe differences in revascularization outcomes between patients with ahDM and nonDM due to limited sample size and the need for MT equipment and techniques improvements.

Polyhedrocytes cells result from the tightening of blood clots driven by platelet contraction accompanied by compaction of RBCs, gradually changing their shape from biconcave to polyhedral [12]. Platelet activation is necessary for clot contraction [38]. It requires platelet cytoskeletal motility proteins and fibrin as the substrate for the contraction of bridging platelets to generate the necessary forces to segregate platelets/fibrin from RBCs and compress these cells into a tightly packed array [12]. Activated platelets may underpin the higher polyhedrocyte content in clots among patients with AIS with a history of ahDM.

Clot permeability (also referred to as perviousness) is the degree to which blood can flow through clot structures. Clot perviousness is considered a key predictor of treatment responsiveness. When treated with intravenous thrombolysis [39] and mechanical thrombectomy [40], pervious clots are correlated with better recanalization outcomes. Further, thrombus perviousness correlates with histologic composition. A recent study by Benson et al. using MSB staining to differentiate platelets from fibrin revealed a higher RBC component and lower fibrin fraction in pervious thrombi than in impervious clots [41]. This study demonstrated that clots in patients with AIS and ahDM were less permeable and had more fibrin and fewer RBC components, consistent with Benson et al. The characteristics of activated platelets and polyhedrocytes in patients with ahDM permit minimization of the space between cells, resulting in more compact and stable clots, and less deformable and permeable. A previous study demonstrated that the objective of clot contraction was to produce a good hemostatic seal and restore blood flow [42]. In contrast, this thrombus characteristic can negatively affect patients with AIS. Relatively porous clots may allow residual arterial flow and retain a degree of oxygenation to downstream tissues [43]. Here, clot contraction may have adverse effects, such as affecting local blood flow and thrombotic permeability of fibrinolytic enzymes, thereby reducing the internal fibrinolysis rate.

This study has several limitations. Laboratory evaluations of hemoglobin A1c and oral glucose tests were not performed. The absence of HbA1c could put some patients in another group. Further, information on medications used by patients with diabetes for blood glucose control was not collected in detail. In this regard, insulin sensitizers (such as pioglitazone and metformin) may help to reduce PAI-1 levels or platelet activity by improving insulin sensitivity. In addition, patients with undiagnosed T2DM may have been classified as non-DM patients, resulting in selection bias. The patients already spontaneously (or through thrombolysis) recanalized were excluded from the study, limiting the patient’s collective and strength of conclusions.

## Conclusion

In conclusion, we demonstrated the effects of ahDM on thrombus composition and contraction-induced RBC deformation. Ischemic stroke severity was affected by ahDM and was associated with poorer functional outcomes. Further, ahDM affected the composition and ultrastructure of clots, and clots from patients with AIS and ahDM exhibited impervious characteristics. Knowledge of the composition and contraction of cerebral thrombi may help improve and predict the effectiveness of thrombectomy or thrombolytic recanalization of occluded vessels and facilitate the development of novel treatment approaches.

## Data Availability

All data generated or analyzed during this study are included in this published article.

## References

[1] Mozaffarian D (2015). Heart disease and stroke statistics--2015 update: a report from the American Heart Association. Circulation.

[2] Luitse MJ (2012). Diabetes, hyperglycaemia, and acute ischaemic stroke. Lancet Neurol.

[3] Tziomalos K (2014). Type 2 diabetes is associated with a worse functional outcome of ischemic stroke. World J Diabetes.

[4] Gray CS (2007). Glucose-potassium-insulin infusions in the management of post-stroke hyperglycaemia: the UK glucose insulin in stroke trial (GIST-UK). Lancet Neurol.

[5] Martini SR, Kent TA (2007). Hyperglycemia in acute ischemic stroke: a vascular perspective. J Cereb Blood Flow Metab.

[6] Kosiborod M (2018). Hyperglycemia in acute coronary syndromes: from mechanisms to prognostic implications. Endocrinol Metab Clin N Am.

[7] Lemkes BA (2010). Hyperglycemia: a prothrombotic factor?. J Thromb Haemost.

[8] Sporns PB (2017). Ischemic stroke: what does the histological composition tell us about the origin of the thrombus?. Stroke.

[9] Simons N (2015). Thrombus composition in acute ischemic stroke: a histopathological study of thrombus extracted by endovascular retrieval. J Neuroradiol.

[10] Choi MH (2018). Erythrocyte fraction within retrieved thrombi contributes to thrombolytic response in acute ischemic stroke. Stroke.

[11] Lam WA (2011). Mechanics and contraction dynamics of single platelets and implications for clot stiffening. Nat Mater.

[12] Cines DB (2014). Clot contraction: compression of erythrocytes into tightly packed polyhedra and redistribution of platelets and fibrin. Blood.

[13] Tutwiler V (2016). Kinetics and mechanics of clot contraction are governed by the molecular and cellular composition of the blood. Blood.

[14] Carr ME, Krishnaswami A, Martin EJ (2002). Platelet contractile force (PCF) and clot elastic modulus (CEM) are elevated in diabetic patients with chest pain. Diabet Med.

[15] Gresele P (2003). Acute, short-term hyperglycemia enhances shear stress-induced platelet activation in patients with type II diabetes mellitus. J Am Coll Cardiol.

[16] Ye G (2020). The role of diabetes mellitus on the thrombus composition in patients with acute ischemic stroke. Interv Neuroradiol.

[17] Erratum. Classification and diagnosis of diabetes. Sec. 2 (2016). Diabetes Care.

[18] Khismatullin RR (2020). Quantitative morphology of cerebral thrombi related to Intravital contraction and clinical features of ischemic stroke. Stroke.

[19] Adams HP (1993). Classification of subtype of acute ischemic stroke. Definitions for use in a multicenter clinical trial. TOAST. Trial of org 10172 in acute stroke treatment. Stroke.

[20] Zaidat OO (2013). Recommendations on angiographic revascularization grading standards for acute ischemic stroke: a consensus statement. Stroke.

[21] Stritt M, Stalder AK, Vezzali E (2020). Orbit image analysis: an open-source whole slide image analysis tool. PLoS Comput Biol.

[22] Kiers L (1992). Stroke topography and outcome in relation to hyperglycaemia and diabetes. J Neurol Neurosurg Psychiatry.

[23] Jørgensen H (1994). Stroke in patients with diabetes. The Copenhagen stroke study. Stroke.

[24] Karapanayiotides T (2004). Stroke patterns, etiology, and prognosis in patients with diabetes mellitus. Neurology.

[25] Tuttolomondo A (2008). Diabetic and non-diabetic subjects with ischemic stroke: differences, subtype distribution and outcome. Nutr Metab Cardiovasc Dis.

[26] Singer DE, Moulton AW, Nathan DM (1989). Diabetic myocardial infarction. Interaction of diabetes with other preinfarction risk factors. Diabetes.

[27] MacDougall NJ, Muir KW (2011). Hyperglycaemia and infarct size in animal models of middle cerebral artery occlusion: systematic review and meta-analysis. J Cereb Blood Flow Metab.

[28] Grant PJ (2007). Diabetes mellitus as a prothrombotic condition. J Intern Med.

[29] Ajjan RA (2013). Diabetes is associated with posttranslational modifications in plasminogen resulting in reduced plasmin generation and enzyme-specific activity. Blood.

[30] Borsey DQ (1984). Platelet and coagulation factors in proliferative diabetic retinopathy. J Clin Pathol.

[31] Pretorius L (2018). Platelet activity and hypercoagulation in type 2 diabetes. Cardiovasc Diabetol.

[32] Undas A (2008). Hyperglycemia is associated with enhanced thrombin formation, platelet activation, and fibrin clot resistance to lysis in patients with acute coronary syndrome. Diabetes Care.

[33] Przygodzki T, et al. Diabetes and hyperglycemia affect platelet GPIIIa expression. Effects on adhesion potential of blood platelets from diabetic patients under in vitro flow conditions. Int J Mol Sci. 2020;21(9):3222. 10.3390/ijms21093222 PMC7247361 32370146

[34] Winocour PD (1994). Platelets, vascular disease, and diabetes mellitus. Can J Physiol Pharmacol.

[35] Elalamy I (2008). Circulating platelet-leukocyte aggregates: a marker of microvascular injury in diabetic patients. Thromb Res.

[36] Singh P, Kaur R, Kaur A (2013). Clot composition and treatment approach to acute ischemic stroke: the road so far. Ann Indian Acad Neurol.

[37] Gunning GM (2018). Clot friction variation with fibrin content; implications for resistance to thrombectomy. J Neurointerv Surg.

[38] Tutwiler V (2021). Pathologically stiff erythrocytes impede contraction of blood clots. J Thromb Haemost.

[39] Mishra SM (2014). Early reperfusion rates with IV tPA are determined by CTA clot characteristics. AJNR Am J Neuroradiol.

[40] Mokin M (2021). Clot perviousness is associated with first pass success of aspiration thrombectomy in the COMPASS trial. J Neurointerv Surg.

[41] Benson JC (2020). Clot permeability and histopathology: is a clot's perviousness on CT imaging correlated with its histologic composition?. J Neurointerv Surg.

[42] Muthard RW, Diamond SL (2012). Blood clots are rapidly assembled hemodynamic sensors: flow arrest triggers intraluminal thrombus contraction. Arterioscler Thromb Vasc Biol.

[43] Santos EM (2016). Thrombus permeability is associated with improved functional outcome and recanalization in patients with ischemic stroke. Stroke.

